# A New Approach to Power Efficiency Improvement of Ultrasonic Transmitters via a Dynamic Bias Technique

**DOI:** 10.3390/s21082795

**Published:** 2021-04-15

**Authors:** Kyeongjin Kim, Hojong Choi

**Affiliations:** Department of Medical IT Convergence Engineering, Kumoh National Institute of Technology, 350-27 Gumi-Daero, Gumi 39253, Korea; 20196092@kumoh.ac.kr

**Keywords:** ultrasonic transmitter device, power amplifier, dynamic bias technique

## Abstract

To obtain a high-quality signal from an ultrasound system through the transmitter, it is necessary to achieve an appropriate operating point of the power amplifier in the ultrasonic transmitter by applying high static bias voltage. However, the power amplifier needs to be operated at low bias voltage, because a power amplifier operating at high bias voltage may consume a large amount of power and increase the temperature of the active devices, worsening the signal characteristics of the ultrasound systems. Therefore, we propose a new method of increasing the bias voltage for a specific period to solve this problem by reducing the output signal distortion of the power amplifier and decreasing the load on the active device. To compare the performance of the proposed method, we measured and compared the signals of the amplifier with the proposed technique and the amplifier only. Notably, improvement was achieved with 11.1% of the power added efficiency and 3.23% of the total harmonic distortion (THD). Additionally, the echo signal generated by the ultrasonic transducer was improved by 2.73 dB of amplitude and 0.028% of THD under the conditions of an input signal of 10 mW. Therefore, the proposed method could be useful for improving ultrasonic transmitter performance using the developed technique.

## 1. Introduction

**Figure 1:**
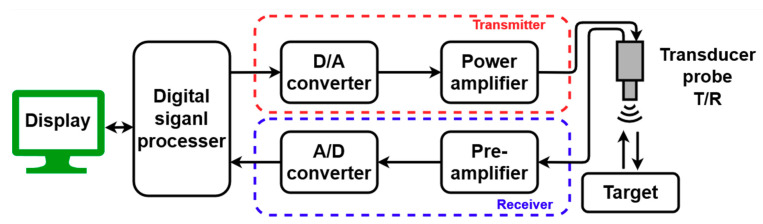
An ultrasonic (ultrasound) system.

An ultrasound (ultrasonic) system can be represented as a block diagram, as shown in Figure 1 [1,2,3]. The input signal in an ultrasound system is amplified through the power amplifier in the ultrasound (ultrasonic) transmitter. The output signal of the transmitter vibrates the piezoelectric element of the transducer probe to transmit ultrasonic waves [4,5]. The signal transmitted through the transducer probe is reflected or attenuated as it passes through the target and is received again through the transducer probe [6]. Because the received signal has a very small amplitude, it needs to be amplified through a pre-amplifier device and then displayed as an image on the computer display after processing the signal [7,8,9].

In an ultrasound system composed as shown in Figure 1, the transmitter is an important factor in determining the image quality [10,11]. This is because the input signal must be linearly amplified to form the desired output signal. However, the variety of variables in the power amplifier design for ultrasonic transmitters needs to be considered because the linearity and efficiency of the power amplifier performance parameters have a trade-off relationship with each other [12]. For example, signal distortion occurs when the bias voltage of the gate of the active device in the power amplifier is lower than the threshold voltage; thus, the drain–source channel of the active device is disconnected for a certain period, thus generating signal distortions from the power amplifiers [13,14,15]. To minimize such signal distortion, bias voltage higher than the threshold voltage is required so that the DC voltage of the gate side of the active device can be maintained to be higher than the threshold voltage [16]. Thus, the distortion of the output signal can be minimized by increasing the operating point of the active device with higher bias voltage [13,17,18].

Like radio frequency power amplifier applications, amplifiers used in ultrasonic transmitters also have a trade-off design relationship with several characteristics, including linearity, efficiency, and bandwidth [3,19,20,21]. The bandwidth of the amplifiers used for typical ultrasonic imaging applications needs to be wide to achieve the high axial resolution, and linearity of the amplifiers needs to be achieved to achieve the appropriate echo signal amplitudes of the ultrasonic transducer [3]. Good linearity of the amplifiers is preferrable for ultrasound harmonic imaging applications because of unwanted harmonic information caused by the target [22,23].

A linear amplifier with high bias voltage is used to increase the ultrasound signal quality or ultrasound image resolution [23]. A linear amplifier, such as a Class A amplifier, has high static bias voltage at the gate of the active device. Therefore, because the drain–source channel of the active device is always formed, switching noise does not exist. Switching noise refers to a phenomenon in which the formed drain–source channel of the active device is suddenly shorted, significant amount of current flows, and a high-frequency ring is generated by the inductance of the substrate wiring or choke inductor [24,25]. However, high bias voltage widens the drain–source channel of the active device and consumes more DC power [26]. For a Class A amplifier, the active device continues to operate regardless of the input signal, pulse width, and input signal amplitude. Additionally, in an ultrasound system, a burst wave with a constant period is used; therefore, the active device operates even during the period in which there is no input signal; hence, more DC power is consumed. Consequently, a large amount of current flowing through the drain source can increase the temperature of the active devices, lowering the performance of the active devices or breaking the active devices.

A Class AB amplifier was used for 1.54 MHz high-power ultrasonic transducers [22]. It had sufficiently high bias voltage, but the operating point of the active device was lower than that of a Class A amplifier. Therefore, the bias voltage may be lower than the threshold voltage of the active device owing to the voltage swing of the input signal [19,26]. In this case, the drain–source channel can be closed, and the output signal can be distorted. An amplifier with high efficiency was proposed for low-power portable ultrasound applications [27]. One of such amplifiers with high efficiency is a Class C amplifier, which uses low bias voltage and has an operating point lower than the threshold voltage [19,26,28]. A Class C amplifier is highly efficient because the operating time of the active device is very short. However, there is very high signal distortion with a narrow bandwidth [29]. A linearized Class C amplifier was developed for a 25 MHz ultrasonic transducer [28]. A Class E amplifier was developed for a 32 MHz capacitive-type ultrasonic transducer [30]. It improved the efficiency by using harmonic components [31]. Although the linearity was low, high efficiency and high output power could be achieved [24,32]. A Class D amplifier was developed to deliver high power to a low frequency power piezoelectric load [33]. Due to the characteristics of Class D amplifier, signal distortion occurs during the amplification process. Because signal distortion does not cause a fatal problem for low frequency power piezoelectric loads, high efficiency of the class D amplifier was achieved.

In the case of an ultrasonic transducer, an additional signal processing method was proposed for the amplifier. In a Class B amplifier, the operating point of the active device is adjusted so that the current flows only during the half cycle of the input signal [34]; thus, high efficiency can be achieved; however, half of the signal is distorted. To improve the distorted signal in a Class B amplifier, a feedback technique is used to increase the linearity of the amplifier [34].

Dynamic bias techniques to improve signal distortions have been used in wireless communication applications. A method using dynamic bias voltage increases the bias voltage at a specific time, thus improving the output signal quality of an amplifier [35,36]. Our proposed method could be utilized irrespective of amplifier topology and it is used for an ultrasonic transducer application using burst wave inputs.

To the best of our knowledge, there are no studies on dynamic bias techniques for power amplifier research in ultrasound applications. Therefore, we propose for the first time a method that increases the bias voltage whenever an input signal is applied. The above method could be attractive for ultrasound systems that mainly use burst waves during a constant period. This is because the bias voltage is increased only during a certain period in which nonlinear characteristics occur. The power consumption can be minimized during the operating off period for the active device such that an input signal is not generated during the operating off period. The proposed dynamic bias technique for a power amplifier was customized for ultrasonic transducers. Consequently, we designed a new type of amplifier by reducing the signal distortion and minimizing the power consumption.

Section 2 explains the principle and circuit operation of the dynamic bias technique for the power amplifier used in an ultrasonic transmitter. Section 3 describes the experimental measurement process of the amplifier used for the ultrasonic transmitter. In Section 4, the measured results of the manufactured power amplifier with and without our proposed method are compared; additionally, the transmitted/received signals through the transducer probe are compared. The study is concluded in Section 5.

## 2. Dynamic Bias Technique for Ultrasonic Transmitters

By increasing the voltage level while applying a square pulse to the input signal, the drain–source channel of the active device can be formed or widened only when there is an input signal [35,36]. Through this dynamic bias technique, a signal can be amplified without applying static bias voltage. However, one must consider the effects of the switching noise generated when the drain source of the active device is turned on. Very high dynamic bias voltage can produce more high-frequency rings owing to variations in the flow of a large amount of current and internal parasitic inductances of the electronic component interconnection [24,25]. Considering the switching noises with linearity and efficiency performances of the amplifier, it becomes necessary to apply an appropriate static bias voltage and a dynamic bias voltage to the power amplifier for an ultrasonic transmitter.

**Figure 2:**
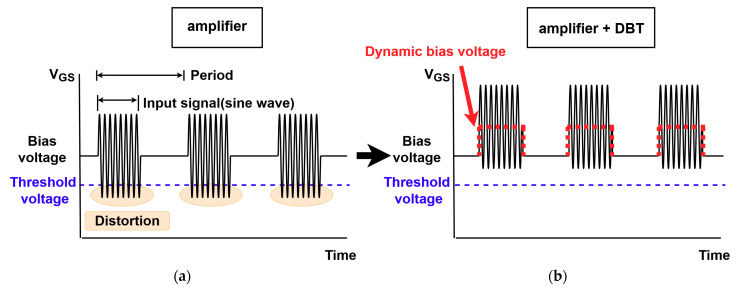
Input gate signal applied to the active device in a power amplifier in which the input signal is applied at certain bias voltage. (**a**) The input gate signal in an amplifier and (**b**) input gate signal at dynamic bias voltage in an amplifier.

Figure 2a shows the input signals applied to the gate of the active device by coupling the burst wave input signal and static bias voltage (V_GS_). This is an example of a typical input gate signal at the active device of an amplifier. As shown in Figure 2a, when V_GS_ falls below the threshold voltage while the input signal operates during a certain period, the drain–source channel is opened, causing signal distortion. To solve this problem, a method of applying dynamic bias voltage, as shown in Figure 2b, was used. Figure 2b shows the signal applied to the gate of the active device when dynamic bias voltage is added to the case in Figure 2a. By generating an additional square pulse to the amplifier, there are no input signals that fall below the threshold voltage. Consequently, an output waveform with less signal distortion can be generated.

**Figure 3:**
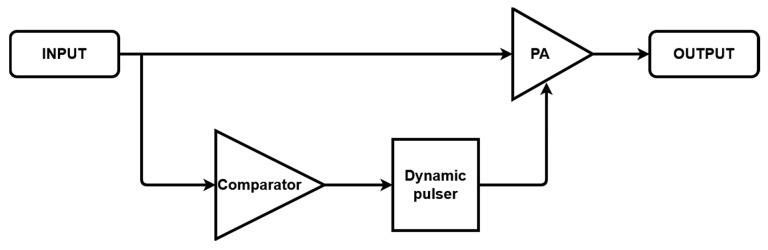
Amplification process using the dynamic bias technique.

Figure 3 shows the amplification process based on the dynamic bias technique. When an input signal is applied, a logic high signal is produced through the comparator. The dynamic pulser calculates the time delay and pulse width when the logic high signal arrives, and a pulse signal is applied to the gate of the active device in the power amplifier. In a dynamic pulser, custom tuning can be performed over wider frequency ranges in the microcontroller unit (MCU). The electrical elements used for the comparator, dynamic pulser, and power amplifier must be properly tuned to be used for some transducers while considering the frequency and amplitude characteristics.

**Figure 4:**
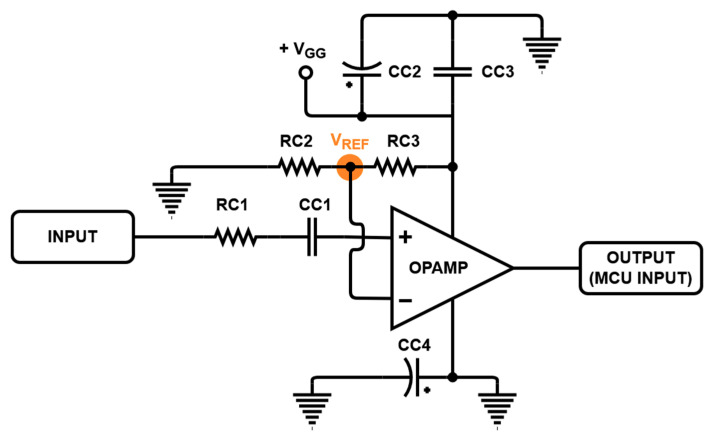
Designed comparator circuit.

Figure 4 shows the comparator circuit, which is also shown in Figure 3. The comparator was constructed using the operational amplifier (OPA847, Texas Instrument Inc., Dallas, TX, USA). The voltage swing of the input signal may be lower than the trigger voltage of the MCU. In this case, the MCU does not detect the input signal so it cannot be performed as digital logic. Therefore, a comparator was composed with the operational amplifier. Because the input impedance of the operational amplifier (OPAMP) is very low, the amplitude of the input signal is distributed between the power amplifier and comparator, and the input signal applied to the gate in the power amplifier can be lowered. Additionally, the pulse signal from the dynamic pulser has certain frequency characteristics, and a feedback route can be formed through the capacitor to block the DC signal used in the amplifier; thus, the distributed signal is minimized and the feedback route is blocked by using CC1 and high values of RC1. RC2 and RC3 are appropriately selected to have the reference voltage V_REF_ to ensure stable operation and prevent oscillation [37]. V_GG_ is used to provide stable DC voltage used in the comparator, dynamic pulser, and gate of the power amplifier. An electrolytic capacitor (CC2) and a ceramic capacitor (CC3) are used in parallel to remove the unwanted noise [38,39]. Consequently, when a sine wave is applied, the logic high signal is fed to the input of the MCU of the dynamic pulser through the comparator. Table 1 presents the values of the resistor and capacitor elements of the comparator circuit shown in Figure 4.

**Figure 5:**
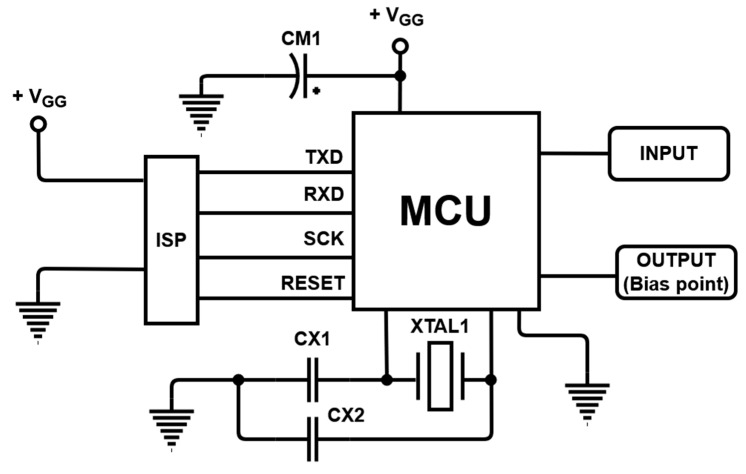
Designed dynamic pulser circuit.

Figure 5 shows the dynamic pulser circuit, which is also shown in Figure 3. The TXD, RXD, SCK, and RESET ports of the MCU, connected to the ISP, are used to communicate with a computer, and they were used to insert the programming codes. An electrolytic capacitor (CM1) was used to supply stable DC voltage to the MCU [40]. The input signal of the MCU of the dynamic pulser comes from the output of the comparator. When a logic high signal is applied as an input, a pulse with a time delay is applied to the power amplifier. Because the time delay by the digital logic of the MCU and the analog circuit of the power amplifier can occur, it becomes necessary to measure the bias point directly through the oscilloscope and tune the components of the amplifiers and MCU units. The output signal of the dynamic pulser is applied to the bias point corresponding to the gate of the active device in the power amplifier. Table 2 presents the values of the crystal and capacitor elements of the dynamic pulser circuit shown in Figure 5.

**Figure 6:**
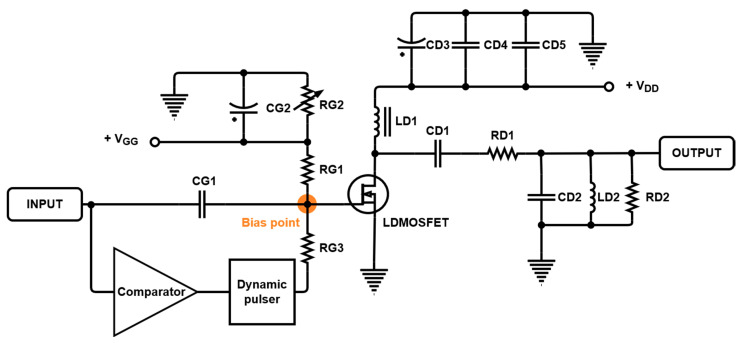
Power amplifier circuit used in the experiment.

Figure 6 shows the designed power amplifier circuit, which is also shown in Figure 3. A laterally diffused metal-oxide semiconductor (LDMOSFET) was used as the active device in the power amplifier. The signal generated from the dynamic pulser had certain frequency characteristics. Accordingly, attenuation and ring-down resulting from capacitive loads can occur due to the parasitic capacitances and inductances of electrical elements [24,41]. Therefore, resistors, rather than capacitors or inductors, were used to prevent static bias voltages from the function generator or comparator. The bias voltage is generated with RG1, RG2, RG3, and internal resistances of the LDMOSFET and MCU. The output (GPIO) of the dynamic pulser is connected through RG3. The An electrolytic capacitor (CD3) and ceramic capacitors (CD4 and CD5) were used to provide stable voltages to the drain of the LDMOSFET. A choke coil inductor (LD1) and CD1 were used to couple and block the signals. Additionally, RD1, RD2, CD2, and LD2 were appropriately adjusted to be compatible with a 20 MHz transducer [42,43]. Table 3 presents the values of the resistor, capacitor, and inductor elements of the power amplifier circuit shown in Figure 6.

## 3. Experimental Environment and Measurement Elements

In power amplifiers, it is necessary to increase the output amplitude while minimizing the signal distortion to obtain appropriate ultrasound image resolution [44]. The higher the output signal generated from the transducer, the higher the sensitivity obtained from the ultrasound system [45]. Therefore, the output amplitudes of the designed power amplifiers were compared with and without the dynamic bias technique. When a signal is amplified through an LDMOSFET, harmonic components that are multiples of the original signal are created. Harmonic components can provide unwanted information compared with the original signal because the attenuation coefficient is medium-dependent [46]. Because it is necessary to minimize the harmonic components of the power amplifier, the total harmonic distortions (THDs) were calculated and compared. A power amplifier consumes most power in the transmitter of an ultrasound system. Therefore, the efficiency of the amplifier must be compared using the power added efficiency (PAE) parameter. Therefore, the PAE was calculated by measuring the DC power and output power to compare the efficiencies of the amplifiers [47]. To analyze the performances of the amplifiers, gain and PAE can be expressed as follows:

(1) $$\mathrm{Gain}\;\left(\mathrm{dB}\right)=20\log\frac{V_{out}}{V_{in}},$$

(2) $$\mathrm{PAE}\;\left(\%\right)=\frac{P_{out}-P_{in}}{DC\;power}\times 100\;\left(\%\right)$$

THD was calculated by measuring the harmonic components up to the fourth order because the harmonic components of the fifth or higher order were very low [19]. One has the following:

(3) $$\mathrm{THD}\;\left(\%\right)=\frac{\sqrt{2nd\;Harmonic^{2}+3rd\;Harmonic^{2}+4th\;Harmonic^{2}}}{fundamental\;signal}\times 100$$

**Figure 7:**
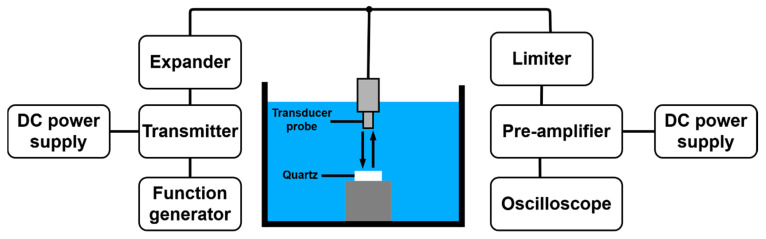
Experimental process for the ultrasonic echo signal test.

In the experiment, the characteristics of the piezoelectric element of the ultrasonic transducer may vary depending on the temperature. To minimize the errors due to temperature variances, a heat sink and cooling fan were used, and a high-frequency cable was used to minimize the signal loss and reflection distortion. Because the output of the amplifier to be examined is very high voltage, a −40 dB attenuator was used to protect the oscilloscope, and a limiter was used while measuring the echo signal. Figure 7 shows the measurement process for the ultrasonic echo signal performance from the ultrasonic transducer (probe). The signal output through the transmitter passes through the expander and is transmitted to the transducer probe [48]. The signal transmitted through the transducer probe is reflected through quartz and returns to the transducer probe. The received signal passes through the limiter, is amplified through a pre-amplifier, and then measured using an oscilloscope [49]. The expander removes the unnecessary noise contained in the output signal generated from the transmitter [50]. A transmitted signal and received signal coexist on the wires between the expander and limiter. The transmitted signal has a very large amplitude, and it can damage the pre-amplifier or oscilloscope; therefore, a limiter is used for protection.

## 4. Results

**Figure 8:**
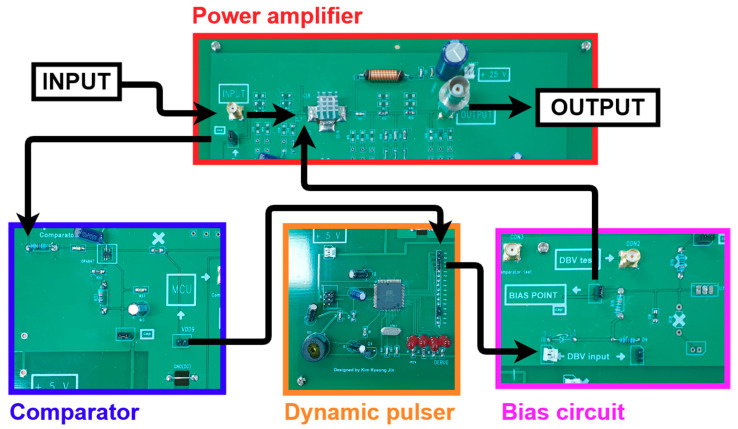
Manufactured power amplifier with the dynamic bias voltage technique.

Figure 8 shows the signal flow of each component in the designed amplifier. Because the power amplifier uses high bias voltage, it exerts a significant amount of load on the LDMOSFET devices [51]. Therefore, the temperature of the LDMOSFET can be increased, and the characteristics of the LDMOSFET can be changed accordingly [52]. Consequently, a heat sink and cooling fan were used to obtain more accurate performances. Additionally, a buzzer and LED were used to debug the programming of the MCU. In the experiment, the input signal was applied through a function generator, and a high-frequency 50-Ω cable was used. Moreover, the output signal was transmitted to the oscilloscope and transducer using a high-frequency 50-Ω cable. There exists a jumper cap between the input signal and comparator, and it can short or open the line. By using the jumper cap to open or short the line, the amplifier only and amplifier using dynamic bias technique (DBT) were examined and compared.

**Figure 9:**
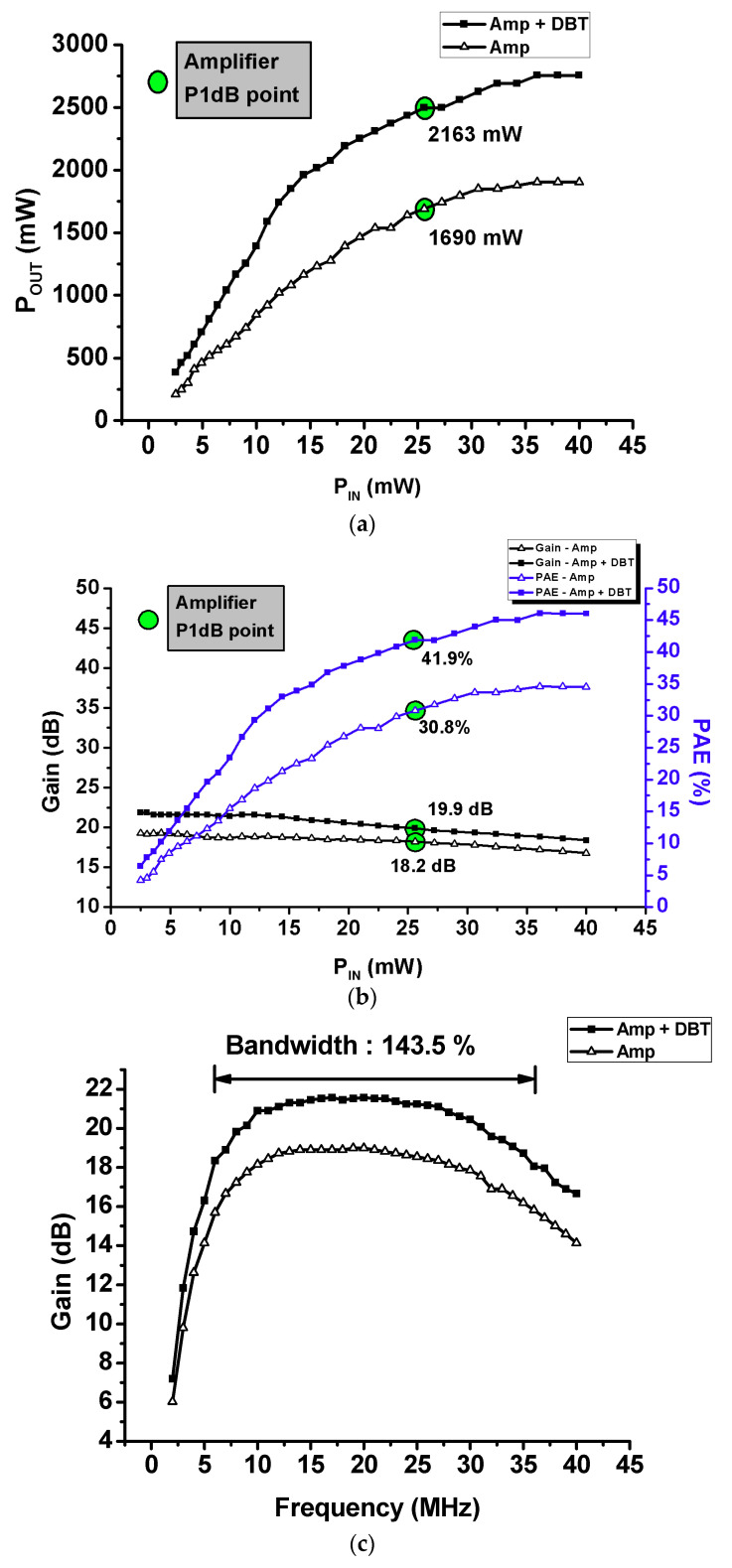
Measured output performances of the amplifier with and without dynamic bias technique (DBT). (**a**) P_OUT_ versus P_IN_, (**b**) gain versus P_IN_, and power added efficiency (PAE) versus P_IN_, and (**c**) gain versus frequency.

Figure 9 shows the measured output power and power gain to compare the amplifier and amplifier using DBT. Figure 9a shows the measured P_OUT_ according to the input signal of the amplifier and amplifier using DBT. Figure 9b shows the measured power gain and PAE of the amplifier and amplifier using DBT. The input signal was measured using a 20-MHz, 1000-cycle burst wave. For the amplifier using DBT, the bias voltage level increases when the input signal is applied; thus, there is less distortion of the input signal, and more current can flow from the drain to source. Additionally, when the static bias voltage increases, a large amount of DC power is consumed owing to the widened drain–source channel in the active device; thus, the PAE can be decreased accordingly. However, the PAE can be improved when an output with high amplitude is formed, and the DC power is minimized by applying a dynamic bias voltage that increases the bias voltage only when the input signal is present. (see Figure 9b). Figure 9c shows the measured gain versus the frequency of the amplifier only and the amplifier with DBT. As shown in Figure 9c, the bandwidth of the designed amplifier only and the amplifier with DBT had the same bandwidth as 143.5%. Expectedly, a higher output, power gain, and PAE can be obtained. When comparing the data between Amp + DBT and Amp in Figure 9, the P_OUT_, power gain, and PAE values of the amplifier using DBT were improved. At the P_1dB_ (1-dB compression) point of the amplifier, the amplifier achieved 1690 mW of P_OUT_, 18.2 dB of gain, 30.8% of PAE, while the amplifier using DBT achieved 2496 mW of P_OUT_, 19.9 dB of gain, and 41.9% of PAE.

**Figure 10:**
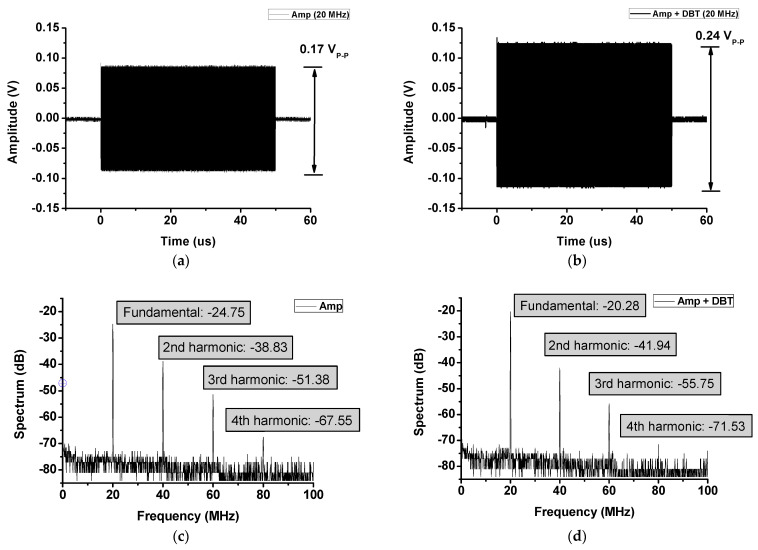
Output waveforms and their fast Fourier transform (FFT) spectrum data of the amplifier only and amplifier using DBT, respectively, with a −40 dB attenuator. (**a**) Output amplitude of the amplifier only. (**b**) Output amplitude of the amplifier using DBT. (**c**) Output FFT spectrum data of the amplifier only. (**d**) Output FFT spectrum data of the amplifier using DBT.

Figure 10a,b show the measured performances with a −40 dB attenuator when an input signal of 20 MHz and 1000-cycle burst wave was applied. Almost no difference in pulse width existed between the measured output performances of the amplifier with and without DBT. However, when DBT was used, the output amplitude of 0.07 V_P-P_ was improved. The graphs of Figure 10c,d are the data using fast Fourier transform (FFT) given from Figure 10a,b data. The FFT spectrum data of the amplifier were −24.75 dB_m_ of the fundamental signal, −38.83 dBm of the second harmonic, −51.38 dBm of the third harmonic and −67.55 dBm of the fourth harmonic components. Additionally, the FFT spectrum data of the amplifier using DBT are −20.28 dB_m_ of the fundamental signal, −41.94 dB_m_ of the second harmonic, −55.75 dB_m_ of the third harmonic and −71.53 dB_m_ of the fourth harmonic components. THDs of the amplifier and amplifier using DBT were 3.91% and 0.68%, respectively. THD was calculated using Equation (3). When using the DBT technique at the amplifier, the 7 V_P-P_ output and 3.23% THD were improved. The harmonic component was mainly attributed to the nonlinearity of the active device. Consequently, Figure 10c,d show that the linearity was increased by using the dynamic bias technique.

**Figure 11:**
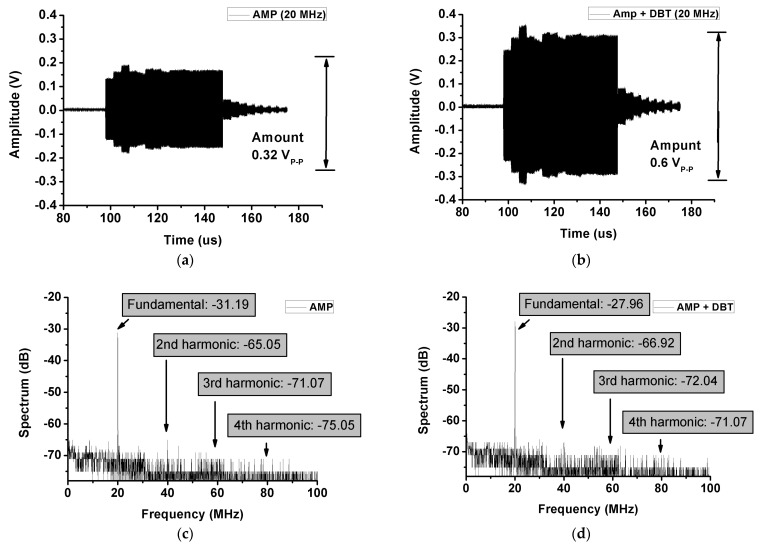
Measured waveform of the echo signal and FFT spectrum data when the amplifier only and amplifier using DBT are used. (**a**) The waveform of the echo signal amplitude when only the amplifier is used. (**b**) The waveform of the echo signal amplitude when the amplifier using DBT is used. (**c**) The echo signal FFT spectrum data when only the amplifier is used. (**d**) The echo signal FFT spectrum data when the amplifier using DBT is employed.

Figure 11 shows the echo signals received through the transducer shown in Figure 7. The received echo signals were passed through a limiter and amplified using a pre-amplifier [53]. The input signal from the power amplifier used a 20-MHz, 1000-cycle burst wave. Figure 11a,b shows the measured waveforms of the echo signal. The amplitude was measured as 0.32 V_P-P_ when the amplifier was used. The amplitude was measured as 0.6 V_P-P_ when the amplifier with DBT was used. The FFT spectrum data were −31.19 dB_m_ at the fundamental signal, −65.05 dB_m_ of the second harmonic, −71.07 dB_m_ of the third harmonic, and −75.05 dB_m_ of the fourth harmonic components. Additionally, the FFT spectrum data of the amplifier using DBT were −27.96 dB_m_ of the fundamental signal, −66.92 dB_m_ of the second harmonic, −72.04 dB_m_ of the third harmonic, and −71.07 dB_m_ of the fourth harmonic components. THDs when using the amplifier only and amplifier using DBT were 0.043% and 0.015%, respectively. The ultrasonic transducer probe worked as a 20-MHz filter. Therefore, the measured harmonic components out of the 20 MHz range were very low. Consequently, when DBT was used in the amplifier, the echo signal increased. Additionally, all the harmonic components decreased (see Figure 11). Consequently, the THD of the ultrasonic echo signal was improved to 0.028%. Therefore, we can conclude that the dynamic bias technique in the amplifier not only improved the output signal and efficiency of the transmitter, but also improved the echo signal.

## 5. Conclusions

To achieve a high-quality signal in an ultrasound system, it is necessary to transmit an output signal with low distortion and large amplitude from the power amplifier to the transducer. For such ultrasound systems, a power amplifier with high bias voltage is generally used. However, a large amount of DC power is consumed, even when there is no input signal generated from the power amplifier with static bias voltage. With the proposed dynamic bias technique, the bias voltage increases only when the input signal operates during a certain period, thus minimizing the wasted DC power while achieving a higher output amplitude. Therefore, in this study, the performances of an amplifier using static bias voltage and an amplifier using a dynamic bias technique were analyzed and compared with each other.

The circuits used to implement the dynamic bias technique comprised a comparator, dynamic pulser, and power amplifier. When an input signal was applied, a logic high signal was applied to the input of the dynamic pulser from the comparator. A dynamic bias voltage was applied to the bias point of the power amplifier by tuning the delay time such that the input signal and pulse signal overlapped at an appropriate time through the MCU in the dynamic pulser. Consequently, the bias voltage increased only when the input signal was present, achieving a high PAE from the distortion-free output signal and minimized DC power consumption. Based on the P_1dB_ of the amplifier, the amplifier achieved 1690 mW of P_OUT_, 18.2 dB of gain, 30.8% of PAE, while the amplifier using DBT achieved 2496 mW of P_OUT_, 19.9 dB of gain, and 41.9% of PAE. By increasing the voltage level at a specific time through dynamic bias voltage, DC power consumption increased compared with when only an amplifier was used, although the output signal increased, thereby increasing the PAE. When the input signal was 10 mW, the amplifier achieved 0.17 V_P-P_ of output amplitude and 3.91% of THD, and the amplifier using DBT achieved 0.24 V_P-P_ of output amplitude and 0.68% of THD. Additionally, the echo signal of the amplifier achieved 0.32 V_P-P_ of amplitude and 0.043% of THD, while the amplifier using DBT achieved 0.6 V_P-P_ of amplitude and 0.015% of THD. Consequently, when dynamic bias voltage was applied at the P_1dB_ point of the amplifier, improvement was achieved with 11.1% of PAE, 1.7 dB of gain and 3.23% of THD. Additionally, the echo signal was improved by 2.73 dB of amplitude and 0.028% of THD at an input signal of 10 mW.

We showed that the THD of the echo signal, as well as the power gain and PAE, was improved by using the dynamic bias technique. The transducer probe of an ultrasound system is designed for various purposes; thus, the power amplifier in the transmitter must be customized to fit the transducer probe. Therefore, an amplifier using the dynamic bias technique can be a new approach that helps improve the resolution of a high-quality signal or image in an ultrasound system and achieve high efficiency transmitters.

## Figures and Tables

**Table 1:** Numerical values of the circuit elements in Figure 4.

Component	Value	Component	Value
RC1	1 kΩ	CC2	100 µF
RC2	680 Ω	CC3	1200 pF
RC3	4.3 kΩ	CC4	10 µF
CC1	1200 pF		

**Table 2:** Numerical values of the circuit elements in Figure 5.

Components	Values	Components	Values
CX1	20 pF	CM1	100 µF
CX2	20 pF	XTAL1	11.0592 MHz

**Table 3:** Numerical values of the circuit elements in Figure 6.

Components	Values	Components	Values
RG1	200 Ω	CD1	1200 pF
RG2	10 kΩ (Potentiometer)	CD2	100 pF
RG3	1.1 kΩ	CD3	220 µF
RD1	51 Ω	CD4	1200 pF
RD2	39 Ω	CD5	100 pF
CG1	1200 pF	LD1	1 µH
CG2	10 µF	LD2	1 µH

## Data Availability

The data presented in this study are included within the article.
